# Detection of SGI1/PGI1 Elements and Resistance to Extended-Spectrum Cephalosporins in *Proteae* of Animal Origin in France

**DOI:** 10.3389/fmicb.2017.00032

**Published:** 2017-01-19

**Authors:** Eliette Schultz, Axel Cloeckaert, Benoît Doublet, Jean-Yves Madec, Marisa Haenni

**Affiliations:** ^1^Infectiologie et Santé Publique, Institut National de la Recherche Agronomique, Université François Rabelais de Tours, UMR 1282Nouzilly, France; ^2^Université Lyon-Agence Nationale de Sécurité Sanitaire de l’Alimentation, de l’Environnement et du Travail, Unité Antibiorésistance et Virulence BactériennesLyon, France

**Keywords:** SGI1, PGI1, *Proteus*, ESBL, AmpC, animal, dog

## Abstract

*Proteae*, and especially *Proteus mirabilis*, are often the cause of urinary tract infections (UTIs) in humans. They were reported as carriers of extended-spectrum β-lactamase (ESBL) genes, and recently of carbapenemases, mostly carried by the *Salmonella* genomic island 1 (SGI1) and *Proteus* genomic island 1 (PGI1). *Proteae* have also lately become an increasing cause of UTIs in companion animals, but antimicrobial susceptibility data in animals are still scarce. Here, we report the characterization of 468 clinical epidemiologically unrelated *Proteae* strains from animals collected between 2013 and 2015 in France. Seventeen *P. mirabilis* strains (3.6%) were positive for SGI1/PGI1 and 18 *Proteae* (3.8%) were resistant to extended-spectrum cephalosporins (ESC). The 28 isolates carrying SGI1/PGI1 and/or ESC-resistance genes were isolated from cats, dogs, and horses. ESBL genes were detected in six genetically related *P. mirabilis* harboring *bla*_V EB-6_ on the SGI1-V variant, but also independently of the SGI1-V, in 3 *P. mirabilis* strains (*bla*_VEB-6_ and *bla*_CTX-M-15_) and 1 *Providencia rettgeri* strain (*bla*_CTX-M-1_). The AmpC resistance genes *bla*_CMY -2_ and/or *bla*_DHA-16_ were detected in 9 *P. mirabilis* strains. One strain presented both an ESBL and AmpC gene. Interestingly, the majority of the ESBL/AmpC resistance genes were located on the chromosome. In conclusion, multiple ESC-resistance genetic determinants are circulating in French animals, even though SGI1-V-carrying *P. mirabilis* seems to be mainly responsible for the spread of the ESBL gene *bla*_VEB-6_ in dogs and horses. These results are of public health relevance and show that companion animals in close contact with humans should be regarded as a potential reservoir of ESC-resistant bacteria as well as a reservoir of ESC-resistance genes that could further disseminate to human pathogens.

## Introduction

*Proteae* are specific *Enterobacteriaceae* comprising bacterial species present in water, soil or in the intestinal tract of humans and animals. In humans, *Proteae*, and especially *Proteus mirabilis*, are often the cause of urinary tract infections (UTIs; Schaffer and Pearson, 2015). They are otherwise opportunistic pathogens responsible for various infections ranging from minor to life-threatening issues. In veterinary medicine, *P. mirabilis* and other *Proteae* are rarely found as pathogens except as a cause of UTIs in companion animals (Bubenik et al., 2007). This pathology is only rarely treated with extended-spectrum cephalosporins (ESC), and the recommended antibiotics are sulphonamides, aminoglycosides, or fluoroquinolones.

*Proteus mirabilis* is naturally susceptible to β-lactams and β-lactamases inhibitors (Stock, 2003). In the late 1990s, the emergence of *P. mirabilis* isolates expressing acquired β-lactamases was first reported in France (Chanal et al., 2000). Extended-spectrum (ESBL) and AmpC β-lactamases are of a critical importance because they both confer resistance to nearly all β-lactams, including ESC. Interestingly, even though the corresponding genes (mostly *bla*_CTX-M-types_ and *bla*_VEB-6_ for ESBL and *bla*_CMY -2_ and *bla*_DHA-16_ for AmpC) are generally located on plasmids which allow an easy intra- or inter-species dissemination, several studies revealed chromosome-encoded ESBL/AmpC genes in *P. mirabilis* (Song et al., 2011; Harada et al., 2012). These chromosome-encoded genes are often carried by genomic islands (such as the *Salmonella* Genomic Island 1, SGI1) or integrating conjugative elements (ICEs) that may also be transmitted (Harada et al., 2010; Mata et al., 2011). While ESBL-producing *P. mirabilis* are nowadays commonly isolated from humans, the first CTX-M-55-producing *P. mirabilis* in animals was only reported in 2011 from a macaque imported from Vietnam to France (Dahmen et al., 2013).

Besides, *P. mirabilis* can also carry various genomic islands conferring multidrug resistance. For example SGI1, the genomic island widely disseminated in *Salmonella*, was first identified in a clinical *P. mirabilis* from a diabetic patient from Palestine in 2006 (Ahmed et al., 2007). SGI1 is a site-specific integrative mobilizable element conferring multidrug resistance initially described in *Salmonella enterica* serovar Typhimurium DT104 (Boyd et al., 2001).

**FIGURE 1:**
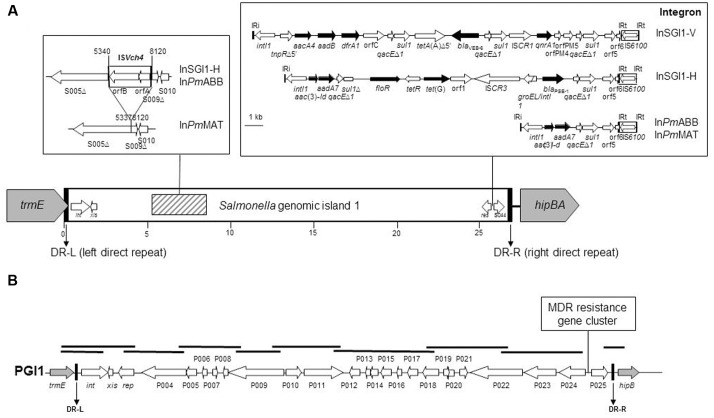
**Schematic view of SGI1 in the *Proteus mirabilis* chromosome.** **(A)** Specific genetic traits of the SGI1 structure, the genetic rearrangement due to IS*Vch4*, and the complex class 1 integrons are represented in frames. Genes and ORFs are shown as arrows, with their orientations of transcription indicated by the arrowheads. Black arrows indicate antibiotic resistance genes. White arrows within boxes represent insertion sequence elements. IRi and IRt are 25-bp imperfect inverted repeats defining the integrase and *tni* ends of complex class 1 integrons. The complex class 1 integrons were drawn from PCR mapping results and GenBank accession numbers AF261825 (SGI1), HQ888851 (SGI1-V), AY458224 (SGI1-H), and JX121638 (SGI1-*Pm*ABB). **(B)** Schematic view of PGI1 in the *P. mirabilis* chromosome. PCRs carried out to map the PGI1 backbone and junctions with the chromosome are indicated by thick black bars. Gray and white arrows represent the chromosomal genes and the backbone of the island, respectively.

*Salmonella* genomic island 1 is the first MDR genomic island identified in *S. enterica*, and contains a complex class 1 integron, named In104 (Hall, 2010). Since the identification of SGI1 in *S.* Typhimurium DT104, more than 30 different SGI1 variants carrying different combinations of antimicrobial resistance genes were described so far (Hall, 2010). The complex In104 integron variants classically possess one or two cassette attachment sites (*attI1*) carrying various resistance gene cassette arrays, contain an IS*6100* element, may contain additional resistance genes, and are bound by 25-bp inverted repeats IRi and IRt (Boyd et al., 2001) (**Figure 1**). In the great majority of these variants, the complex In104 integron or its variants are found always at the same position in the SGI1 scaffold, i.e., between the resolvase gene *res* (also named *tnpR*) and the open reading frame (ORF) S044 at the 3′ end of SGI1 (Hall, 2010). A few additional variations occurred in the SGI1 backbone of some variants, especially the insertion/deletion created by IS*Vch4* between ORF S005 and S009 that was found in several SGI1 variants (SGI1-H, -Ls, -Ks, -Ps, Qs, -*Pm*ABB, and -*Pm*MAT) in *S. enterica* and *P. mirabilis* (Doublet et al., 2008; Siebor and Neuwirth, 2013).

*Salmonella* genomic island 1 is found integrated most of the time within the last 18 bp of the well-conserved chromosomal *trmE* gene (also named *thdF*). SGI1 is specifically mobilized *in trans* by conjugative plasmids of the IncA/C family (Doublet et al., 2005). Only this plasmid family has been shown to be able to mobilize SGI1 (Douard et al., 2010). The main reason of this specificity is that the SGI1 excision from the chromosome is triggered by the master activator AcaDC encoded by IncA/C conjugative plasmids (Carraro et al., 2014; Kiss et al., 2015). Then, as an extrachromosomal form, SGI1 is able to hijack the conjugative apparatus encoded by IncA/C plasmids to be conjugally transferred to a recipient cell (Carraro et al., 2014).

Since 2006, *P. mirabilis* strains carrying different SGI1 variants have only been reported in China and France (Boyd et al., 2008; Siebor and Neuwirth, 2013; Qin et al., 2015). Importantly, the SGI1-V variant, which is specifically found in *P. mirabilis*, harbors the *bla*_VEB-6_ gene and was first reported in a lethal human case in France (Siebor and Neuwirth, 2011).

Recently, a new multidrug resistant genomic island named *Proteus* Genomic Island 1 (PGI1) was described in human *P. mirabilis* isolates in France (Siebor and Neuwirth, 2014). A specific PGI1 variant, PGI1-*Pm*PEL, was shown to harbor both the *bla*_VEB-6_ gene and the carbapenemase-encoding gene *bla*_NDM-1_ (Girlich et al., 2015). *P. mirabilis* isolates of animal origin were also shown to carry SGI1 or PGI1. Indeed, SGI1-positive *P. mirabilis* isolates were reported in poultry and swine farms in China (Lei et al., 2014, 2015). In France, we recently described the very first cases of SGI1 (including the VEB-6-producing SGI1-V variant) or PGI1-positive *P. mirabilis* in dogs (Schultz et al., 2015).

The SGI1/PGI1-positive *P. mirabilis* isolates reported in animals and humans so far were sporadic cases. Considering the apparent emergence of these genetic determinants in *P. mirabilis*, our aim was to investigate the prevalence of SGI1 and PGI1 in *Proteae* of animal origin in France, and to characterize molecularly the collected strains. In line with recent observations that a SGI1-V-carrying *P. mirabilis* clonal population was shared between humans and animals, we also investigated the genetic relationship of those isolates in order to draw hypotheses on a possible transfer between the two populations. Finally, as these islands in *P. mirabilis* isolates were also shown to occasionally capture ESBL or carbapenemase genes, we investigated the global prevalence of those genes in the same collection.

## Materials and Methods

### Bacterial Strains and Antibiotic Susceptibility Testing

Between April, 2013 and February, 2015, a total of 468 clinical non-duplicate *Proteae* isolates (*P. mirabilis*, *n* = 459; *P. vulgaris*, *n* = 1; *P. penneri*, *n* = 1; *Morganella morganii*, *n* = 2; *Providencia rettgeri*, *n* = 4; *P. stuartii*, *n* = 1) were collected from various animal hosts corresponding to distinct and epidemiological unrelated individuals (dogs, *n* = 411; cats, *n* = 25; horses, *n* = 13; bovine, *n* = 7; rabbit, *n* = 3; ovine, *n* = 3; ferret, *n* = 2; snake, *n* = 2; chicken; *n* = 1; bird, *n* = 1) and originating from distant geographical areas throughout France. These isolates were collected through the Resapath, the French antimicrobial resistance surveillance network in animal pathogens^1^. Identification was performed by peripheral veterinary laboratories and then confirmed at the Anses laboratory in Lyon, France, using Api20E strips (bioMérieux, Marcy l’Etoile, France). Isolates were screened for antibiotic resistance by the disk diffusion method according to the guidelines of the CA-SFM with 30 antibiotics of veterinary or human interest^2^.

### SGI1/PGI1 Detection and PCR Mapping

*Salmonella* genomic island 1 detection was performed by PCR amplification with degenerated primers designed in order to amplify all known SGI1 and PGI1 integrase genes (FwintSGI1HR, 5′ATGTTGCGTCAGGCYGAGGC; RvintSGI1HR, 5′GAGTGYCCAAGAAGSCGAGAG). The chromosomal location was confirmed by PCR amplification of the left and right junctions in the chromosome, as previously described (Schultz et al., 2015). The genetic diversity of SGI1 was assessed by PCR mapping covering the entire island using primers previously described (Siebor and Neuwirth, 2011, 2013). PGI1 detection, chromosomal location, backbone mapping and resistance gene detection were performed by PCR, as previously described (**Figure 1**) (Siebor and Neuwirth, 2014).

### Identification and Genetic Location of ESBL and AmpC Genes

β-lactamase genes detection was performed by PCR, as previously described (Dallenne et al., 2010). For the CTX-M-1 group, an additional PCR was performed using external primers (ISEcp1L1, 5′ CAGCTTTTATGACTCG; P2D, 5′ CAGCGCTTTTGCCGTCTAAG) and the amplicons were sequenced.

The chromosomal location of these genes was assessed with the *I-Ceu*I (New England Biolabs, Hertfordshire, UK) technique (Liu et al., 1993). After digestion of the complete DNA, DNA fragments were separated by Pulsed-Field Gel Electrophoresis (PFGE) in TBE 0.5 X at 14°C using a CHEF Mapper (Bio-Rad Laboratories, Richmond, CA, USA). Running conditions were 6 V/cm with a switch time of 5.3–49.9 s for 19.7 h. Southern blots were performed by transferring the DNA on a Hybond-N^+^ membrane and hybridizing the membrane with DIG-labeled probes specific for the 23S rDNA, the ESBL (*bla*_VEB-6_, *bla*_CTX-M-1_, or *bla*_CTX-M-15_) and AmpC (*bla*_CMY -2_ or *bla*_DHA-16_) genes of interest (see detailed primers in **Supplementary Table S1**). Probes were prepared by PCR using labeled DIG-dUTP (PCR DIG probe synthesis kit; Roche Diagnostics, Indianapolis, IN, USA). Detection was performed using the DIG DNA Labeling and Detection Kit (Roche Diagnostics) according to the manufacturer’s instructions.

The plasmidic location of these genes was assessed by Southern blots on S1-PFGE gels (New England Biolabs, Hertfordshire, UK). Running conditions were 6 V/cm with a switch time of 1–30 s for 20 h. Hybridization was performed as described above for Southern blots on *I-Ceu*I gels, using the same ESBL/AmpC probes and the DIG DNA Labeling and Detection Kit.

Finally, all SGI1/PGI1-positive, ESBL and AmpC strains were analyzed by PCR-based replicon typing (PBRT kit; Diatheva, Fano, Italy) in order to type the plasmids carrying the ESBL/AmpC genes.

### Genetic Relationship of the Isolates

The genetic diversity was investigated by PFGE. DNA plugs were digested with *Sma*I (Promega, Madison, WI, USA) and genomic DNA was separated by CHEF Mapper gel electrophoresis in TBE 0.5 X at 14°C. The running conditions were 6 V/cm with a switch time of 5–20 s for 22 h. *Xba*I-digested *S. enterica* serovar Braenderup strain H9812 was used as size ladder. The DNA patterns were analyzed using BioNumerics software version 4.5 (Applied Maths, Sint-Martens-Latem, Belgium) to construct a phylogenetic tree. Analysis was performed using the Dice coefficient with optimization set at 0.5% and tolerance at 1%.

## Results and Discussion

### Prevalence and Molecular Characterization of the SGI1/PGI1 Genomic Islands

**FIGURE 2:**
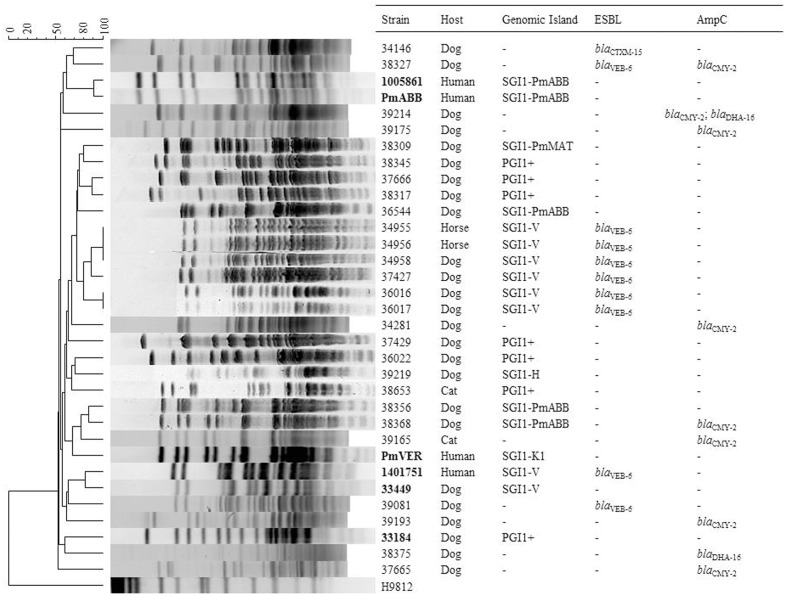
**Analysis of *Sma*I-Pulsed-Field Gel Electrophoresis (PFGE) patterns obtained from the relevant *P. mirabilis* isolates.** PFGE profiles were compared using BioNumerics software version 4.5 (Applied Maths) with settings of 0.5% optimization and 1.0% tolerance. DNA of *S. enterica* serovar Braenderup strain H9812 was used as standard size marker in PFGE experiments and as outgroup in analysis (Hunter et al., 2005). The isolates in bold correspond to previously published profiles included for comparison purposes (Schultz et al., 2015).

*Salmonella* genomic island 1/*Proteus* genomic island 1-carrying *P. mirabilis* are emerging pathogens in humans and animals. In a recent study, SGI1/PGI1-carrying *P. mirabilis* were reported in animals in France but no large-scale non-biased data on their prevalence were available (Schultz et al., 2015). Here, we investigated a large collection of 468 *Proteae* isolates of animal origin in France to estimate the prevalence of the SGI1/PGI1 elements. We also assessed the prevalence of resistance to broad-spectrum cephalosporins in those *Proteae* isolates considering that the *bla*_VEB-6_ gene was recurrently reported on the SGI1-V variant in *P. mirabilis* (Siebor and Neuwirth, 2011; Schultz et al., 2015). Among the 468 isolates studied, 17 *P. mirabilis* (17/468, 3.6%) were positive for SGI1 (11 isolates) or PGI1 (6 isolates) by PCR (**Figure 2** and **Table 1**). They were mostly recovered from dogs (*n* = 13; 13/411, 3.2%), but also from cats (*n* = 2; 2/25, 8%), and horses (*n* = 2; 2/13, 15.4%). While *Proteae* are more frequently isolated from dogs, SGI1/PGI1 elements were more prevalent in cats and horses. This would deserve confirmation due to the low number of isolates collected from these two animal categories.

**Table 1:** Antibiotic resistance profiles of *Proteus mirabilis* strains of interest in this study.

Strain	Isolation date (yyyy/mm/dd)	Host	Geographic area^b^	Pathology	Antibiotic resistance profile^a^
34146	2013/04/17	dog	Haute-Savoie	Urinary tract infection	ESBL; StrKanAprTobNetSss
34381	2013/10/09	dog	Alpes-Maritimes	Urinary tract infection	AmpC; Chl
34955	2013/11/22	horse	Oise	Skin infection	ESBL; KanTobNetChlSssTmpNalEnr
34956	2013/10/25	horse	Calvados	Skin infection	ESBL; StrKanTobNetChlSssTmpNalEnr
34958	2013/07/30	dog	Paris	Urinary tract infection	ESBL; StrKanAmkAprGenTobNetChlSssTmpNalEnr
36016	2013/11/05	dog	Val-de-Marne	Abdominal infection	ESBL; StrKanAmkGenTobNetChlSssTmpNalEnr
36017	2013/22/04	dog	Val-de-Marne	Urinary tract infection	ESBL; StrKanGenTobNetChlSssTmpNalEnr
36022	2013/11/10	dog	Alpes-Maritimes	Urinary tract infection	StrSss
36544	2014/03/05	dog	Paris	Urinary tract infection	StrChlSssTmpNalEnr
37427	2014/04/04	dog	Val-de-Marne	Urinary tract infection	ESBL; KanGenTobNetChlSssTmpNalEnr
37429	2014/03/14	dog	Loire	Otitis	StrKanGenTobChlSss
37665	2014/04/22	dog	Paris	Urinary tract infection	AmpC
37666	2014/03/21	dog	Val-de-Marne	Wound	AmxStrKanGenTobSssTmpNalEnr
38309	2014/09/09	dog	Haute-Savoie	Unknown	StrSssTmpNalEnr
38317	2014/09/09	dog	Tarn	Otitis	AmxStrKanGenTobChlSssTmpNalEnr
38327	2014/09/29	dog	Oise	Skin infection	ESBL + AmpC; StrKanChlSssTmp
38346	2014/07/09	dog	Hauts-de-Seine	Urinary tract infection	AmxStrKanGenTobChlSss
38356	2014/07/15	dog	Aisne	Otitis	StrChlSssTmpNalEnr
38368	2014/08/21	dog	Loire-Atlantique	Urinary tract infection	AmpC; ChlSssTmpNalEnr
38375	2014/08/21	dog	Côtes-d’Armor	Otitis	AmpC; Chl
38653	2014/11/03	cat	Seine-St-Denis	Urinary tract infection	StrKanGenTobChlSss
39081	2014/12/01	dog	Val-de-Marne	Urinary tract infection	ESBL; StrKanAmkGenTobNetChlSssTmpNalEnr
39165	2014/10/02	cat	Loire-Atlantique	Urinary tract infection	AmpC; SssTmpNalEnr
39175	2014/10/31	dog	Val-de-Marne	Otitis	AmpC; AprChlNal
39193	2014/10/09	dog	Paris	Otitis	AmpC; StrChlTmpNal
39214	2014/11/18	dog	Val-de-Marne	Skin infection	AmpC; AprChlNal
39219	2015/01/26	dog	Somme	Unknown	StrChlSssTmpNalEnr
39465^c^	2014/09/19	cat	Gironde	Unknown	ESBL; SssTmpNalEnr


^a^*ESBL, extended-spectrum beta-lactamase; Amx, amoxicillin; Chl, chloramphenicol; Ffc, florfenicol; Amk, amikacin; Apr, apramycin; Gen, gentamicin; Tob, tobramycin; Net, netilmicin; Kan, kanamycin; Str, streptomycin; Sss, sulfonamides; Tmp, trimethoprim; Nal, nalidixic acid; Enr, enrofloxacin*. ^b^*French department: The department is the administrative subdivision of France*. ^c^P. rettgeri *strain*.

Among the 11 SGI1-positive *P. mirabilis*, six showed an ESBL phenotype using the double disk synergy test by antibiogram. PCR mapping of the SGI1 resistance gene cluster and backbone revealed that all six ESBL-positive isolates harbored the SGI1-V variant carrying the *bla*_VEB-6_ gene (**Figure 1**). In the five other SGI1-positive *P. mirabilis* isolates, PCR mapping of the antimicrobial resistance gene cluster gave positive results for different complex class 1 integrons, i.e., InSGI1-H, In*Pm*ABB and In*Pm*MAT (**Figures 1, 2** and **Table 1**). PCR mapping of the SGI1 backbone confirmed that SGI1 elements with InSGI1-H, In*Pm*ABB, and In*Pm*MAT harbored the same 2789 bp deletion at position 5340-8120 (GenBank accession number AF261825) in the region spanning from ORF S005 to ORF S009, as previously described. Except for SGI1-In*Pm*MAT, this deletion was replaced by the insertion of IS*Vch4* (also called IS*1359*) (**Figure 1**). Interestingly, this region contains two ORFs S007 and S006 coding for homologs of the master activator AcaDC of IncA/C conjugative plasmids and named SgaDC (SGI1 activator, subunits D and C; Poulin-Laprade et al., 2015; Muranyi et al., 2016). The SgaDC activator (also named FlhDC_SGI1_) was shown to be active on the same AcaDC-dependent promoter regions, i.e., P*_xis_*, and thus should be also implicated in transfer and/or maintenance of SGI1 (Muranyi et al., 2016). The absence of *flhDC*_SGI1_ in these variants and the partial deletion of ORF S005 (*traN*) may have implications in their spread that needs to be further studied. In addition to the classical penta-resistance of SGI1 to amoxicillin, chloramphenicol, streptomycin and sulphonamides, other SGI1/PGI1-conferred resistances were amikacin, apramycin, gentamicin, tobramycin, netilmicin, kanamycin, nalidixic acid, trimethoprim, and enrofloxacin.

The six PGI1-positive *P. mirabilis* isolates were characterized by PCR mapping of the whole backbone. PCR products of the expected sizes were obtained for the complete mapping indicating a conserved PGI1 genetic structure in these isolates (**Figure 1**). All six isolates showed similar antibiotic resistance profiles as the ones previously described (Siebor and Neuwirth, 2014; Schultz et al., 2015). Interestingly PGI1, which was only recently reported in *P. mirabilis*, was represented in more than one-third of the genomic islands characterized here, thus confirming the proportion observed in a recent study on a much smaller number of isolates (Schultz et al., 2015). This may suggest either a previously undetected situation in the *Proteae* population or a recent and rapid spread of PGI1 elements, which were first described in 2014 (Siebor and Neuwirth, 2014). Our data also indicate that the dissemination of SGI1/PGI1 in multidrug-resistant *P. mirabilis* in animals is not a sporadic phenomenon and should be considered with great attention.

### Extended-Spectrum and AmpC β-Lactamases

Eighteen strains (18/468, 3.8%) harbored an ESBL profile (*n* = 9), an AmpC profile (*n* = 8), or a combination of both (*n* = 1) after antibiotic susceptibility analysis. Only one AmpC-producing strain did not present any additional resistance (**Table 1**). Otherwise, proportions of strains resistant to non-β-lactam antibiotics were particularly high for chloramphenicol (14/18, 77.8%), streptomycin (10/18, 55.6%), trimethoprim/sulphonamides (13/18, 72.2%), nalidixic acid (13/18, 72.2%), and enrofloxacin (10/18, 55.6%). Of note, none of the strains were resistant to carbapenems.

In addition to the six SGI1-V positive *P. mirabilis* described above, the ESBL phenotype was detected in four other strains. Two *P. mirabilis* isolates (38327 and 39081) harbored the *bla*_VEB-6_ gene independently of the SGI1 element (**Figure 2**), whereas the two last ESBL-producing isolates harbored the *bla*_CTX-M-15_ gene (*P. mirabilis* 34146) and the *bla*_CTX-M-1_ gene (*P. rettgeri* 39465). The prevalence of ESBL producers was lower than the one recently reported in France. However, the previous sampling was based on isolates conserved by the veterinary laboratories and a bias toward non-susceptible isolates cannot be excluded (Schultz et al., 2015). On the contrary, no ESBL-producing isolate was detected in Japan (Harada et al., 2014). In any case, *Proteae* from animal origin present much less ESC-resistant isolates than those from human origin, since the nowadays ESBL rate in animals is very close to the French situation in human medicine in the late 1990s (Chanal et al., 2000).

Concerning AmpC resistance phenotypes, the *bla*_CMY -2_ gene was detected alone in 7 *P. mirabilis* isolates whereas another *P. mirabilis* isolate (38375) presented a *bla*_DHA-16_ gene (**Figure 2**). Finally, one *P. mirabilis* isolate (39214) possessed both *bla*_CMY -2_ and *bla*_DHA-16_ genes (**Figure 2**). The presence of multiple ESC-resistance genes in two strains (*bla*_VEB-6_/*bla*_CMY -2_ in 38327 and *bla*_CMY -2_/*bla*_DHA-16_ in 39214) shows the capacity of *P. mirabilis* to accumulate redundant resistance genes and thus acting as a potential reservoir.

**FIGURE 3:**
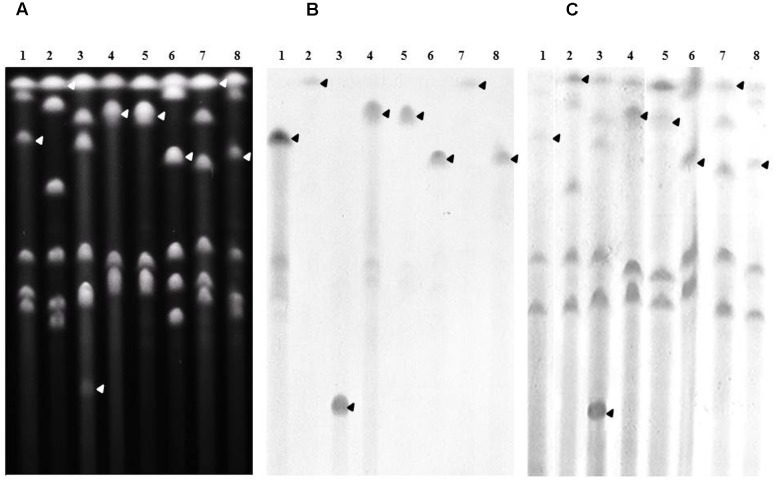
**Chromosomal localization of *bla*_CMY -2_ in the relevant *P. mirabilis* isolates.** **(A)** Whole genomic DNAs of isolates 34381 (lane1), 37665 (lane 2), 38327 (lane 3), 38368 (lane 4), 39165 (lane 5), 39175 (lane 6), 39193 (lane 7), and 39214 (lane 8) were digested with *I-Ceu*I, and the restricted fragments subjected to PFGE. DNA fragments were transferred to a nylon membrane and hybridized with probes specific to *bla*_CMY -2_ **(B)**, and the 23S rRNA gene **(C)**. The arrows indicate the bands of interest.

**FIGURE 4:**
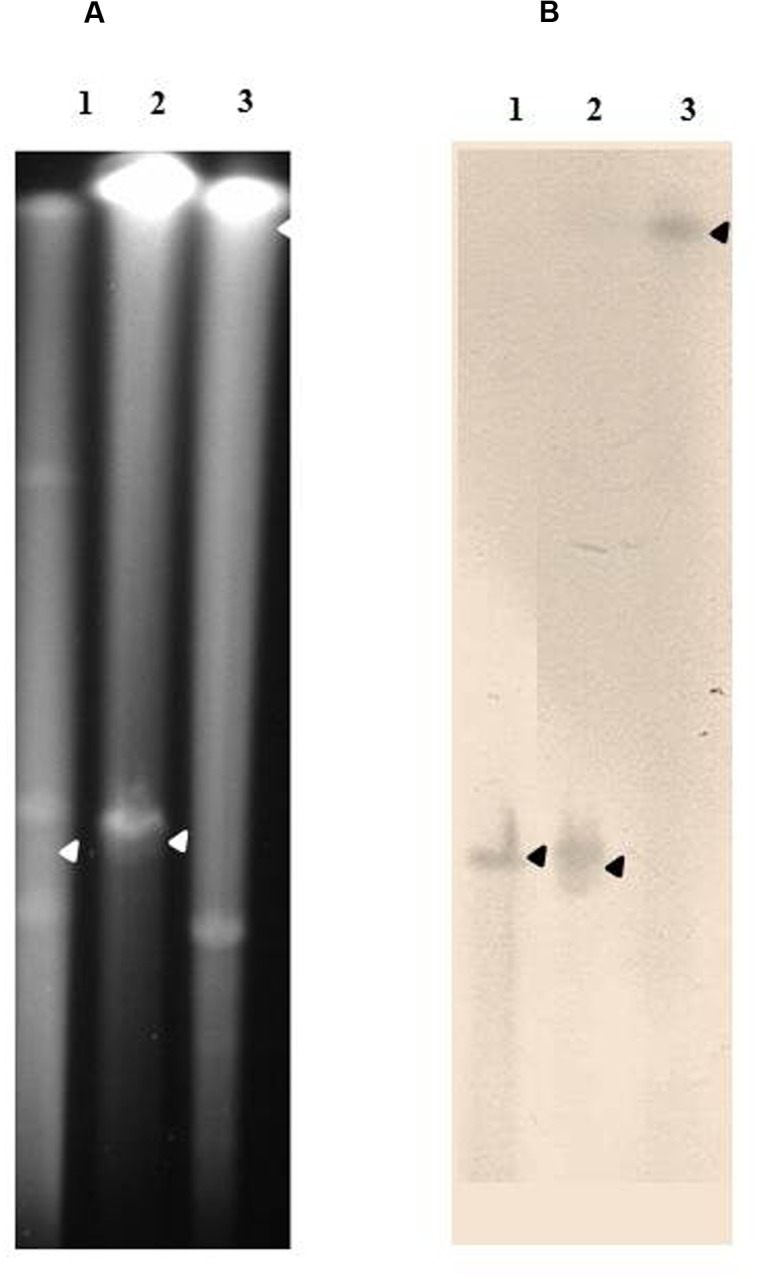
**Plasmidic localization of *bla*_CTX-M-1 group_ genes in the relevant isolates.** **(A)** Whole genomic DNAs of a CTX-M-15-carrying *E. coli* isolate used as a positive control (lane 1), *P. mirabilis* isolate 34146 (lane 2), and *P. rettgeri* isolate 39465 (lane 3), were digested with S1-nuclease, and the restricted fragments subjected to PFGE. DNA fragments were transferred to a nylon membrane and hybridized with a probe specific to *bla*_CTX-M_ **(B)**. The arrows indicate the bands of interest.

The chromosomal location of genes was assessed by Southern-blots on *I-Ceu*I-PFGE using the probes corresponding to the ESBL/AmpC genes carried by the studied isolates, as well as probes specific for the 23S rDNA. This method revealed that all *bla*_CMY -2_ (**Figure 3**) and *bla*_DHA-16_ genes (**Supplementary Figure S1**) were located on the bacterial chromosome. All *bla*_VEB-6_ genes carried by the SGI1-V variant as well as the *bla*_VEB-6_ gene identified in isolate 39081 were also proved to be encoded by the chromosome (**Supplementary Figure S2**). On the contrary, the plasmidic localization of genes was proved by Southern-blots on S1-nuclease-PFGE using adequate probes. The last non-chromosomal *bla*_VEB-6_ (38327; **Supplementary Figure S3**) and *bla*_CTX-M-1_ (*P. rettgeri* 39465; **Figure 4**) genes were thus shown to be carried on plasmids, which were considered as non-typable because of the total absence of amplification using the PBRT kit. Finally, the *P. mirabilis* isolate 34146 carried two copies of the ESBL gene *bla*_CTX-M-15_, one located on the chromosome and the other on a non-typable plasmid (**Figure 4**). Whereas ESBL genes were mostly reported on plasmids in *Enterobacteriaceae*, it is noteworthy to observe that it is seemingly not the case in *Proteae*. These data strongly suggest that *Proteae* may be more prone than other *Enterobacteriaceae* to integrate resistance determinants into the chromosome, mostly on SGI1/PGI1 or other genomic islands.

### Genetic Diversity of the Isolates

*Sma*I PFGE revealed that all SGI1/PGI1-positive isolates were genetically unrelated as were the ESBL/AmpC producing isolates (**Figure 2**), except the six SGI1-V positive isolates that belonged to the same cluster but interestingly differed from the ones previously reported (Schultz et al., 2015). This clustering suggests the spread of a clonal population among different and unrelated individuals. Such a dominance of a clonal population of SGI1-V/*bla*_VEB-6_ *P. mirabilis* strains in humans and companion animals may result from close contacts between these two populations, as also demonstrated for the transfer of other multidrug resistant bacteria or plasmids, such as those carrying ESBL genes. Indeed, the transmission of bacterial clones from animals to humans (or vice-versa) through physical contacts or contact with contaminated saliva or feces has been described (Espinosa-Gongora et al., 2015; Ljungquist et al., 2016).

## Conclusion

In this study, we showed a significant prevalence rate (∼4%) of ESBLs/AmpC in *P. mirabilis* of animal sources. A long-term survey is now needed to decipher whether these are emerging phenotypes or only sporadic cases. The multidrug resistance genomic islands SGI1 and PGI1 play a major role in the dissemination of ESBLs/AmpC genes as well as other non-β-lactam resistance genes. Moreover, we report the spread of the SGI1-V/*bla*_VEB-6_-carrying *P. mirabilis* clonal population to horses. This peculiar ESBL-producing *P. mirabilis* population was previously recognized in humans and dogs and highly suspected in poultry isolates (Seiffert et al., 2013). Altogether, these data suggest an inter-transmission pathway of public health relevance that needs further investigations to be clarified. Therefore, *P. mirabilis* should be regarded as a potential reservoir of resistance traits in companion animals making us believe that veterinarians should pay more attention to *P. mirabilis* as an opportunistic multidrug-resistant pathogen.

## Author Contributions

ES, MH, J-YM, BD, and AC designed the experiments. ES did the experiments. ES, MH, and BD analyzed the data. ES and MH drafted the manuscript. BD, J-YM, and AC actively contributed to the manuscript’s writing. All authors approved the final version of this manuscript.

## Conflict of Interest Statement

The authors declare that the research was conducted in the absence of any commercial or financial relationships that could be construed as a potential conflict of interest.

## Footnotes

1 www.resapath.anses.fr

2 www.sfm-microbiologie.org
