# Supplementary material for: Detection of SGI1/PGI1 Elements and Resistance to Extended-Spectrum Cephalosporins in Proteae of Animal Origin in France
Source: Front Microbiol. 2017 Jan 19;8:32. doi: 10.3389/fmicb.2017.00032 (PMC5243843; doi:10.3389/fmicb.2017.00032)
Supplement: Supplementary file 1 [file Table_1.DOCX]

**Supplementary data**

**Table S1.** Primers used for preparing the Southern blot probes

| Primer | | Nucleotide sequence (5'-3') | | Region amplified | |
| --- | --- | --- | --- | --- | --- |
| 23S-for  23S-rev  Veb-Ext-Up | AATGATGGCCAGGCTGTCTCC  CCGCCGTCGATATGAACTCTTG  ATTTAACCAGATAGGAGTACA | | 23S rRNA  *bla*_VEB_ | |  |
| Veb-Ext-Low | CGGTTTGGGCTATGGGCAG | |  |  |  |
|  |  | |  | |  |
| MA_1 | SCSATGTGCAGYACCAGTAA | | *bla*_CTXM (all groups)_ | |  |
| MA_2 | CCGCRATATGRTTGGTGGTG | |  |  |  |
|  |  | |  | |  |
| MulticaseDHA-for | TGATGGCACAGCAGGATATTC | | *bla*_DHA_ | |  |
| MulticaseDHA-rev | GCTTTGACTCTTTCGGTATTCG | |  |  |  |
|  |  | |  | |  |
| CF1 | ATGATGAAAAAATCGATATG | | *bla*_CMY_ | |  |
| CF2 | TTATTGCAGTTTTTCAAGAATG | |  |  |  |
