# Supplementary material for: Detection of SGI1/PGI1 Elements and Resistance to Extended-Spectrum Cephalosporins in Proteae of Animal Origin in France
Source: Front Microbiol. 2017 Jan 19;8:32. doi: 10.3389/fmicb.2017.00032 (PMC5243843; doi:10.3389/fmicb.2017.00032)
Supplement: Supplementary file 2 [file Image_1.PDF]

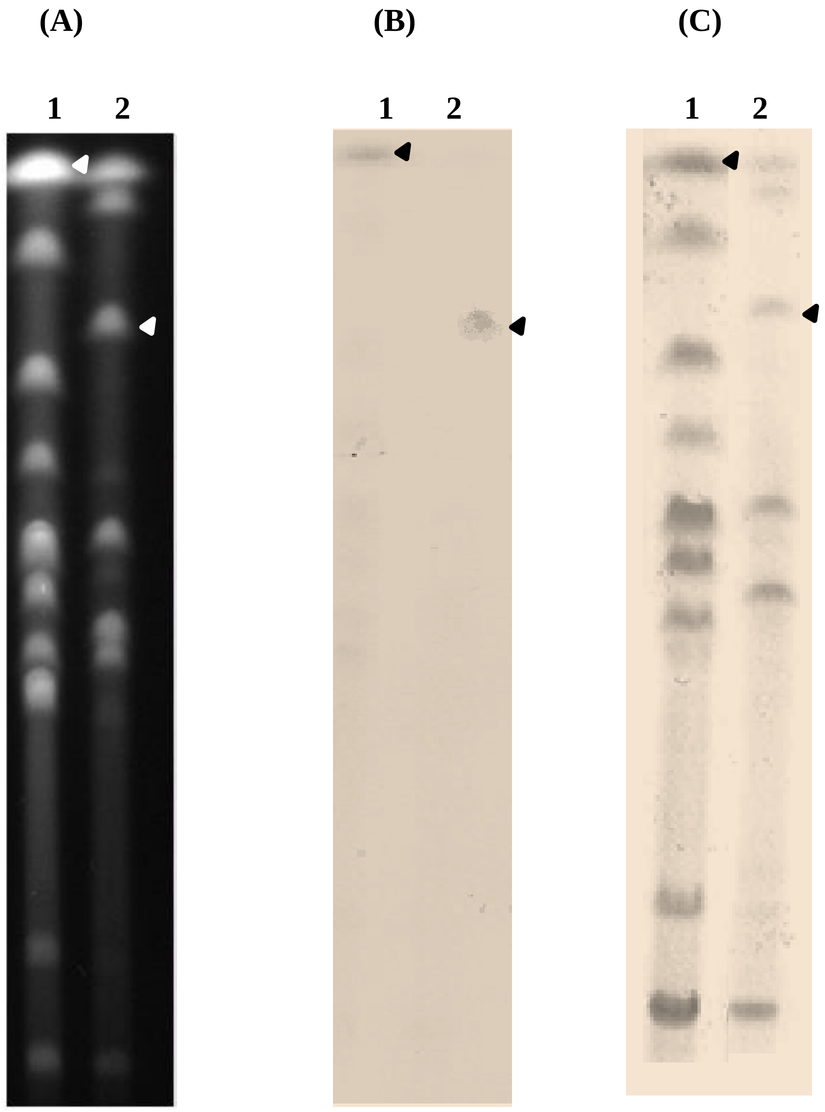

Figure S1. Chromosomal localization of *bla*$_{DHA-16}$ in the relevant *P. mirabilis* isolates.
(A) Whole genomic DNAs of isolates 38375 (lane1) and 39214 (lane 2) were digested with *I-Ceu*I, and the restricted fragments subjected to pulsed-field gel electrophoresis. DNA fragments were transferred to a nylon membrane and hybridized with probes specific to *bla*$_{DHA}$ (B), and the 23S rRNA gene (C). The arrows indicate the bands of interest.

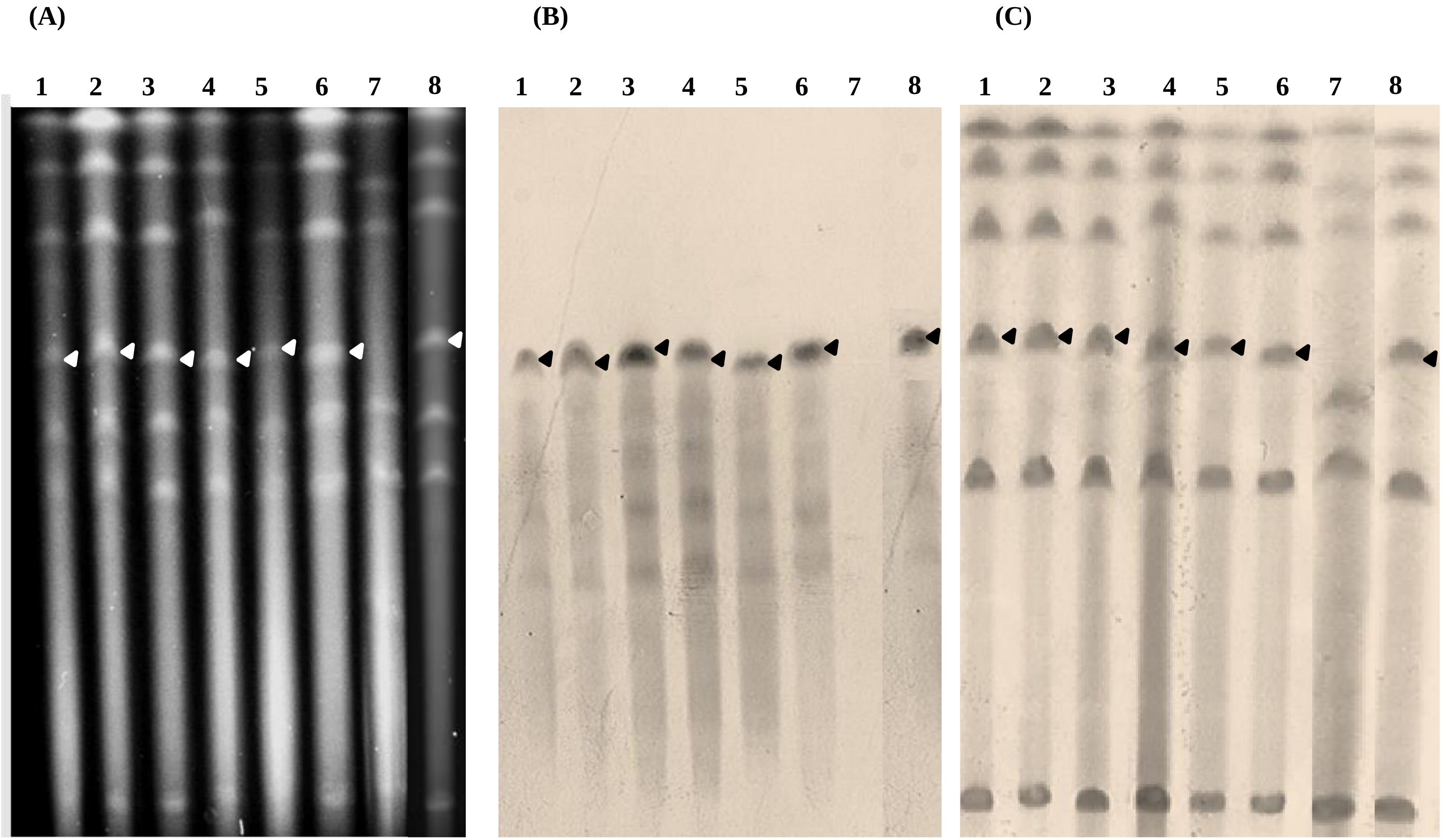

Figure S2. Chromosomal localization of *bla*$_{\text{VEB-6}}$ in the relevant *P. mirabilis* isolates.

(A) Whole genomic DNAs of isolates 34955 (lane 1), 34956 (lane 2), 34958 (lane 3), 36016 (lane 4), 36017 (lane 5), 37427 (lane 6), 38327 (lane 7) and 39081 (lane 8) were digested with *I-Ceu*I, and the restricted fragments subjected to pulsed-field gel electrophoresis. DNA fragments were transferred to a nylon membrane and hybridized with probes specific to *bla*$_{\text{VEB-6}}$ (B), and the 23S rRNA gene (C). The arrows indicate the bands of interest. The absence of *bla*$_{\text{VEB-6}}$ hybridization for isolate 38327 (lane 7) proves that this enzyme is not chromosomally-encoded.

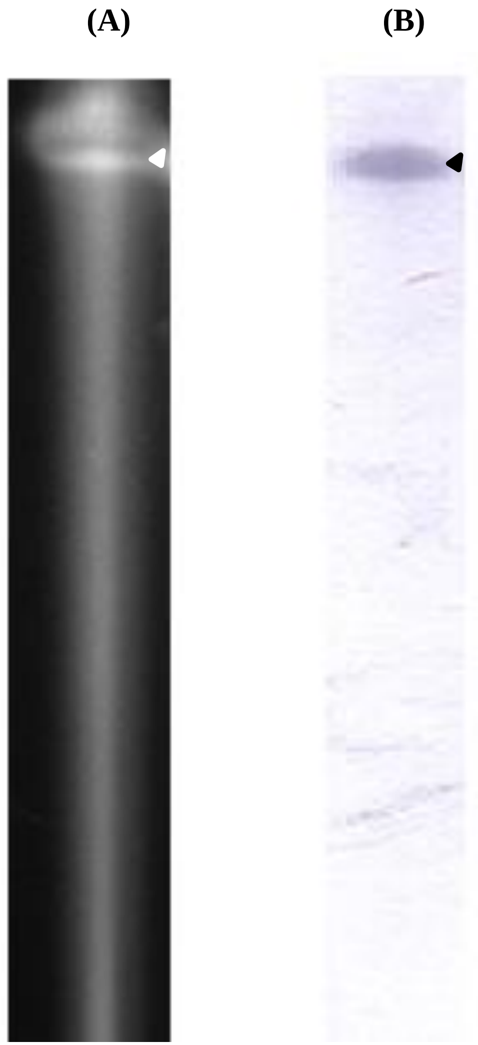

Figure S3. Plasmidic localization of *bla*$_{\text{VEB-6}}$ in the *P. mirabilis* isolate 38327.
(A) Whole genomic DNAs of the isolate *38327* was digested with S1-nuclease, and the restricted fragments subjected to pulsed-field gel electrophoresis. DNA fragments were transferred to a nylon membrane and hybridized with probes specific to *bla*$_{\text{VEB-6}}$ (B). The arrows indicate the bands of interest.
